# Synthesis and Modification of Hydroxyapatite Nanofiber for Poly(Lactic Acid) Composites with Enhanced Mechanical Strength and Bioactivity

**DOI:** 10.3390/nano11010213

**Published:** 2021-01-15

**Authors:** Han-Seung Ko, Sangwoon Lee, Jae Young Jho

**Affiliations:** School of Chemical and Biological Engineering, Seoul National University, Seoul 08826, Korea; khs88@snu.ac.kr (H.-S.K.); tttyyy0403@snu.ac.kr (S.L.)

**Keywords:** hydroxyapatite nanofiber, poly(lactic acid), polymer composite, polymer grafting, mechanical properties

## Abstract

To enhance the bioactivity of poly(lactic acid) (PLA), a potential bone repair material, without the lowering of mechanical strength, hydroxyapatite (HA) was introduced in the form of nanofibers as the filler for application in spinal implant materials. HA nanofibers (HANF) with aspect ratio as high as ~100 were synthesized by controlling the starting pH of the reaction. While the tensile and flexural strength of PLA/HANF composites were enhanced compared with those of PLA resin, and were higher for the composites with HANF of higher aspect ratio. To further strengthen the composites, HANF was grafted with PLA chain to form HANF-g-PLA, which could improve the interface between the HANF and matrix PLA. PLA/HANF-g-PLA composites showed even higher tensile and flexural strength than PLA/HANF composites, apparently due to the better dispersion and interfacial adhesion. The composite containing 10 wt% HANF-g-PLA showed the flexural strength of 124 MPa, which was 25% higher than that of PLA resin. In the bioactivity test using a simulated body fluid solution, the rate and uniformity of the apatite growth were observed to be higher for the composites with HANF, and were even higher for those with HANF-g-PLA. This study suggested the possibility of using the PLA/HANF-g-PLA composite in the field of spinal implant materials.

## 1. Introduction

Metals like titanium and magnesium have been the most popular material for spinal implants by virtue of their superior mechanical strength and processability. Some disadvantages, however, have been pointed out for metal implants; image distortion in postoperative observations, stress shielding effect on the bone around the implant, and the need of additional surgery for removal [1,2]. These problems could be resolved when a polymeric material, especially a biodegradable polymer, was employed instead of metallic material. The biodegradable polymers considered for biomedical applications have been poly(lactic acid) (PLA), poly(glycolic acid) (PGA), and polycaprolactone (PCL) [3]. Biodegradable polymers did not show the problems with biocompatibility required as an implant material [4,5]. The biodegradation time of PCL was more than 24 months, which was longer than that of PLA and PGA [6]. It has been considered an enough time for the bones to be regenerated. However, the application of PCL to spinal implant materials has been limited because the mechanical strength of PCL was much lower than that of human spine bone [7]. PGA has exhibited similar mechanical properties to engineering plastic like polyetheretherketone [8,9]. However, implants based on PGA could be degraded before sufficient repair of bone because the biodegradation rate of PGA was higher than that of PLA and PCL [10]. The balance between mechanical properties and biodegradation rate of PLA was better than that of other biodegradable polymers, so researches to apply PLA to spinal implant materials have been conducted [11]. However, PLA’s mechanical strength including flexural strength required for spinal implants was lower than that of human spine bone. Additionally, PLA itself did not have the ability to heal bones.

Another desirable property for spinal implants has been bioactivity, the ability of the material to promote the bone regeneration. As bioactivity of PLA itself was low, there have been the efforts to enhance the bioactivity by adding bioactive fillers like bioceramics [12,13,14,15]. Among bioceramics, hydroxyapatite (HA) has been the most general because of its structural and functional similarities to the minerals present in human bones. The addition of HA, however, should result in the drop of mechanical properties. The modulus might increase due to the high modulus of HA, the ultimate properties like tensile and flexural strength should be lowered due to the poor interface between HA and PLA. The agglomeration of HA in the PLA composites has actually been observed during composite fabrication, which made the mechanical properties of PLA/HA composites lower than those of PLA resin [16]. The general approach to improve the interfaces of polymer composites has been the addition of compatibilizer or grafting the polymer chains on the surface of the fillers. The introduction of poly(acrylic acid) as the compatibilizer for HA/PLA composite did not bring significant enhancement of interfacial adhesion and thus mechanical strength [17]. PLA has been grafted onto the surface of HA by various methods including direct graft polymerization and linking polymer with coupling agent [18,19,20]. Despite the improved dispersion of HA and interfacial adhesion between HA and PLA matrix in these efforts, the increase in mechanical properties such as tensile strength and flexural strength were less than 5% compared to those of PLA.

It has been well established that the mechanical properties of polymers could be greatly enhanced by the reinforcement with fibrous fillers [21,22]. It has also been well known that the degree of reinforcement was further enlarged by using the fibers with nanometer-scale dimensions. Like other nanoscale fillers, the reason why nanofibers have been so efficient in reinforcing was the high surface area-to-volume ratio or the high aspect ratio. For example, the tensile strength of polypropylene (PP) composite containing 4 wt% of carbon nanotubes increased by 7% compared to that of PP even without surface treatment [23]. HA has also been synthesized in the form of nanofibers by introducing surfactants or urea [24,25]. When preparing a composite containing dental resin and 10 wt% of hydroxyapatite nanofiber (HANF), the biaxial flexural strength of the composite increased by 22% compared to that of dental resin even without surface modification [26]. Although no cases of manufacturing the composites containing PLA and HANF have been reported, it seemed that the reinforcement of the composite through fiber could be applied to PLA/HANF as in the previous study on dental resin/HANF.

In the present study, we were to report the PLA/HANF composites, in which HANF was grafted with PLA chain. HA was synthesized in the form of nanofiber, and grafted by PLA to improve the interfacial adhesion between HANF and PLA and the dispersion of HANF in the composites. The effect of aspect ratio and grafting of PLA on the mechanical properties and bioactivity of the composites were examined.

## 2. Materials and Methods

### 2.1. Materials

PLA used in this study was commercial product of Natureworks with the trade name of 2003D. The molecular weight of PLA was 159,000 g/mol [27]. Calcium nitrate tetrahydrate (Ca(NO_3_)_2_·4H_2_O), Ammonium phosphate dibasic ((NH_4_)_2_HPO_4_), urea, hexamethylene diisocyanate (HMDI), and dibutyltindilaurate (DBTDL) were purchased from MilliporeSigma (Burlington, MA, USA). Nitric acid (HNO_3_, 70%), N,N-dimethylformamide (DMF), chloroform, and ethanol were purchased from Daejung Chemicals (Gyeonggi-do, Korea).

### 2.2. Synthesis of HANF

To the mixture of 300 mL each of 0.16 M Ca(NO_3_)_2_·4H_2_O and 0.1 M (NH_4_)_2_HPO_4_ aqueous solution, 0.5 M HNO_3_ was added until the desired pH was reached. After 300 mL of 0.5 M urea aqueous solution was added, the temperature was raised to 95 °C and maintained for 120 h. The reaction was cooled to room temperature, and the produced HA nanofibers were filtered. HANF was washed with distilled water for five times and ethanol for two times to completely remove the residual ions and water. The final product was dried at 70 °C for 24 h in a vacuum oven (Jeiotech, OV-11, Daejeon, Korea).

### 2.3. Surface Grafting of PLA on HANF

HANF (2 g) in DMF (100 mL) was sonicated and stirred under nitrogen at room temperature. HMDI (0.4 mL, 2.50 mmol) and DBTDL (15.8 mg, 1 mol% of HMDI) were added to the suspension. The reaction continued with stirring at room temperature for 6 h. After the reaction, HMDI-grafted HANF (HANF-HDMI) was separated by centrifugation at 10,000 rpm and washed with DMF for three times and chloroform for two times to completely remove the reactants and solvent. The HANF-HMDI was dried at 50 °C for 24 h in a vacuum oven.

HANF-HMDI (2 g) in chloroform (100 mL) was sonicated at room temperature. PLA with molecular weight 5000 and 14,600 was synthesized in advance by referring to a study [28]. Synthesized PLA (6 g) in chloroform (100 mL) was stirred at room temperature. The HANF-HMDI suspension was added into the PLA solution, and the mixture was stirred under nitrogen protection for 1 h. DBTDL (7.6 mg and 2.5 mg, respectively, 1 mol% of PLA) was added to the mixture, and the reaction continued with stirring at room temperature overnight. After the reaction, PLA-grafted HANF-HMDI (HANF-g-PLA) was separated by centrifugation at 10,000 rpm and washed with chloroform for five times to completely remove the free PLA that did not graft on the surface of HANF-HMDI. The HANF-g-PLA was dried at 50 °C for 24 h in a vacuum oven.

### 2.4. Preparation of PLA/HANF Composites

The PLA/HANF and PLA/HANF-g-PLA composites were prepared as follows. Pre-weighed dried HANF or HANF-g-PLA was uniformly suspended in 70 folds (in weight) chloroform with sonication and stirring. And the suspension was added into 5 wt% PLA/chloroform solution to achieve the HANF content of 5, 10, and 15 wt% in the composites. The mixtures were precipitated in an excess of ethanol, and the composites were dried in a vacuum-oven at 50 °C for 24 h to remove the residual solvent.

### 2.5. Characterization and Measurement

X-ray diffraction (XRD): The crystal structure and crystallinity of HANF were analyzed by XRD (Bruker, D8 Advance, Billerica, MA, USA) with Cu Kα radiation and 2*θ* range from 10 to 60°.

Fourier transform-infrared (FT-IR): The chemical structures of each fillers were analyzed by FT-IR (Bruker, TENSOR27, Billerica, MA, USA) spectroscopy with an attenuated total reflection accessory in the range from 4000 to 500 cm^−1^.

X-ray photoelectron spectroscopy (XPS): The chemical bonds of each fillers were analyzed by XPS (ThermoFisher Scientific, K-alpha+, Waltham, MA, USA) over the wide scanning energy range from 0 to 1350 eV with a pass energy of 200 eV and the high sensitivity spectrum of N1s and P2p with a pass energy of 40 eV.

Thermogravimetric analysis (TGA): The amount of grafted materials on the HANF surface was determined by TGA (TA Instruments, Discovery TGA, New Castle, DE, USA). The measurements were performed under nitrogen gas flow at a rate of 20 °C/min from room temperature to 100 °C, an isothermal process at 100 °C for 10 min, and a rate of 10 °C/min from 100 °C to 700 °C.

Field emission scanning electron microscope (FE SEM): The morphological analysis of the composites was examined using FE SEM (Carl Zeiss, SUPRA 55VP, Oberkochen, Germany). The fracture surface was obtained by breaking the specimen after immersion in liquid nitrogen for 1 min.

Mechanical properties measurement: The specimens for measuring tensile properties and flexural properties were prepared using injection molding (Bautek, BA-915A, Gyeonggi-do, Korea). The cylinder and mold temperatures were set at 180 °C and room temperature, respectively. The time required for injection molding was between 10 and 15 min for each specimen. All specimens for mechanical properties measurement were annealed at 100 °C for 2 h, then cooled to room temperature for 24 h to reduce thermal stress and to improve crystallinity. The tensile properties of the composites were determined using universal testing machine (UTM, Lloyd, LR10K, West Sussex, UK) at 25 °C and at the crosshead speed of 1 mm/min according to ASTM D638 type V. The flexural properties of the composites were determined using the UTM at 25 °C and at the crosshead speed of 2 mm/min according to ISO 178.

Bioactivity test: thin plate-shaped specimens prepared in the same manner as the above injection molding process were soaked in 5 times concentration of simulated body fluid (5×SBF) solution at 37 °C for one, three, and five days. A series of brief evacuation-repressurization cycles were carried out to force the solution into the pores of the composites until no air bubbles emerged from the composite surface. At the end of each soaking period, specimens were washed thoroughly with distilled water, dried at 50 °C for 48 h in a vacuum oven, and weighed. Three specimens were prepared for each experiment, and the average value of them was used.

## 3. Results and Discussion

### 3.1. Structure of HANF

**Figure 1:**
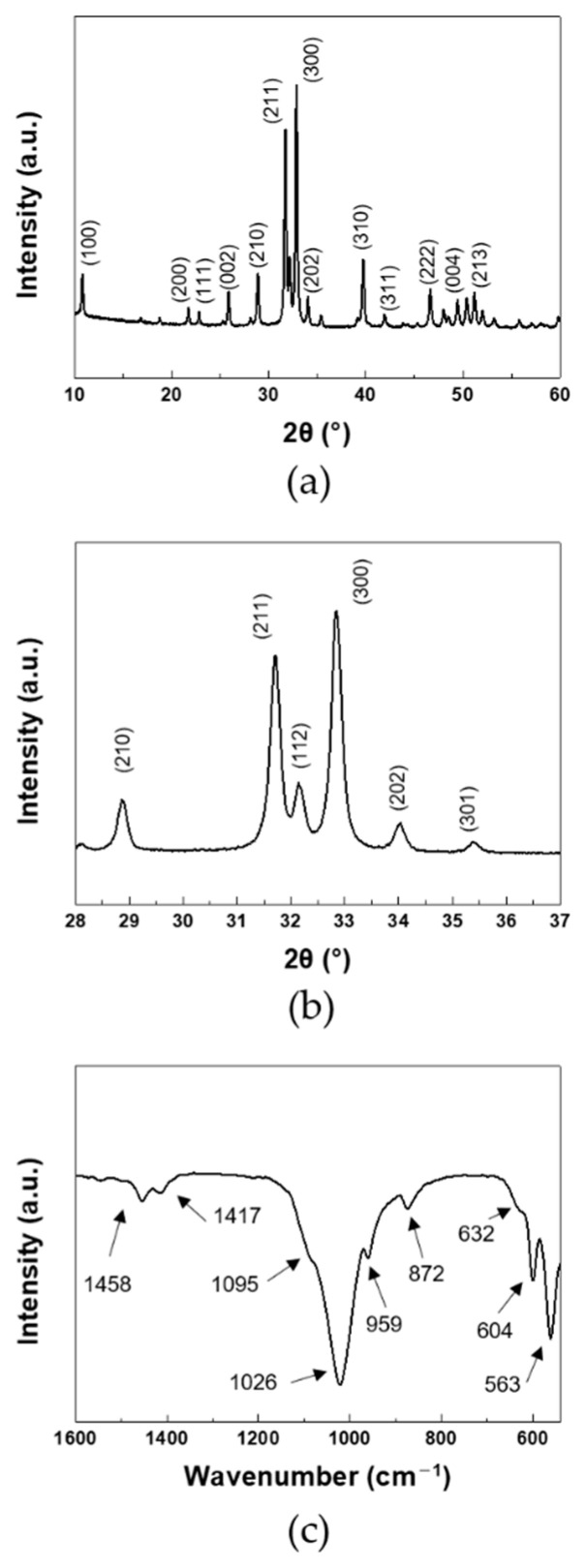
(**a**,**b**) XRD, and (**c**) FT-IR of HANF: (**b**) magnified region of 28–37°.

In Figure 1, XRD and FT-IR spectra of products were investigated to identify the chemical structure of the synthesized products. In XRD, all of the diffraction peaks were coincided with the pure hydroxyapatite phase. The same bands were observed for the synthesis of HA [29]. The crystallinity of the synthesized HA was calculated by following equation [30]:

$$X_{c}\;=\;1-\;\left(V_{112/300}/I_{300}\right)$$

where *I*_300_ was the intensity of (300) reflection and *V*_112/300_ was the intensity of the hollow between (112) and (300) reflections. The crystallinity of the synthesized HA was 92%. The XRD patterns showed that the well-crystallized HA was achieved. The bands in FT-IR agreed with the reported FT-IR data for the carbonated HA [31]. The peaks at 1095 and 1026 cm^−1^ were attributed to the asymmetric stretching of PO_4_. The symmetric stretching of PO_4_ appeared at 959 cm^−1^. The peaks at 604 and 563 cm^−1^ were attributed to the in-plane bending of PO_4_. The peaks at 632 cm^−1^ corresponded to the liberation of OH^−^. With the incorporation of carbonate anions, the spectra exhibited the peaks at 1458 and 1417 cm^−1^ due to the symmetric stretching of A type and B type CO_3_, respectively. The bending of CO_3_ appeared at 872 cm^−1^. The same bands were observed for the AB-type carbonated HA [32]. Hence, the synthesized product was AB-type carbonated HA.

As the pH of the solution increased with urea decomposition, the phase of calcium phosphate preferentially formed at each pH was different [33]. dicalcium phosphate anhydrate (DCPA, CaHPO_4_) and octacalcium phosphate (OCP, CaH_2_(PO_4_)_6_) were preferentially formed at pH 2.4–3.1 and 3.8–5.2, respectively. When the pH increased to 6.2–6.5, DCPA and OCP, which had been previously produced, were hydrolyzed to HA ((Ca_10_(PO_4_)_6_(OH)_2_)) to form HANF. The formation and phase change of calcium phosphate could be represented by the following equations [33]:

$${\mathrm{Ca}}^{2+}+{\;\mathrm{HPO}}_{4}{}^{2-}\;\to {\;\mathrm{CAHPO}}_{4}\;\left(\mathrm{DCPA}\right)\;10{\mathrm{CaHPO}}_{4}+2H_{2}O\;\to {\;\mathrm{Ca}}_{10}{\left({\mathrm{PO}}_{4}\right)}_{6}{\left(\mathrm{OH}\right)}_{2}\;\left(\mathrm{HA}\right)\;+4H_{3}{\mathrm{PO}}_{4}$$

$$8{\mathrm{Ca}}^{2+}+8{\mathrm{HPO}}_{4}{}^{2-}\;\to {\;\mathrm{Ca}}_{8}H_{2}{\left({\mathrm{PO}}_{4}\right)}_{6}\;\left(\mathrm{OCP}\right)\;+2H_{3}{\mathrm{PO}}_{4}\;5{\mathrm{Ca}}_{8}H_{2}{\left({\mathrm{PO}}_{4}\right)}_{6}+8H_{2}O\;\to 4{\mathrm{Ca}}_{10}{\left({\mathrm{PO}}_{4}\right)}_{6}{\left(\mathrm{OH}\right)}_{2}\;\left(\mathrm{HA}\right)\;+6H_{3}{\mathrm{PO}}_{4}$$

The shapes of DCPA and OCP were known as plate-like and blade-like, respectively [34]. One unit cell of OCP could be hydrolyzed to two unit cells of HA due to their similar structure [35]. The morphology of HANF formed from OCP could be maintained in blade-like morphology by the epitaxial overgrowth of OCP, resulting in an increase in the aspect ratio of the synthesized HANF [35]. However, epitaxial overgrowth of OCP was disturbed by the fusion of DCPA to HANF. DCPA acted as an unstable part of OCP growth [33]. In order to obtain high aspect ratio HANF, the amount of DCPA formed in the solution must be minimized.

**Figure 2:**
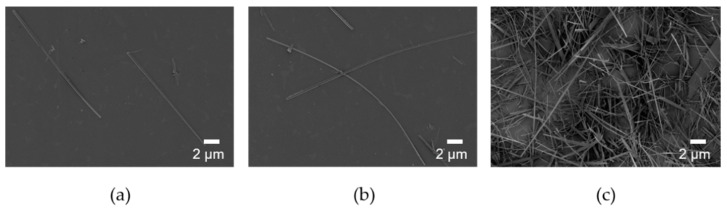
SEM images of HANF with different starting pH: (**a**) pH 2.2, (**b**) and (**c**) pH 2.7.

The starting pH of the reaction increased from 2.2 to 2.7 to reduce the amount of DCPA preferentially formed in the solution at low pH. The morphology of the synthesized HA was shown in Figure 2. The width of the synthesized HANF was ~300 nm in both cases. The number of nucleation sites was not changed significantly because the concentration of the reactants was the same. In a study, the morphology of the synthesized HA was similar even when HA was synthesized with different temperature and reaction time at the same concentration [29]. Therefore, in HANF of similar width, the aspect ratio of HANF increased as the length of HANF increased. When the starting pH of the reaction was 2.2 and 2.7, the lengths of the synthesized HANF were 22 μm and 31 μm, respectively. The aspect ratio of HANF increased from 71 ± 6 to 103 ± 13 as the starting pH of the reaction was changed to reduce the formation of DCPA. The mechanical strength of the short fiber composites theoretically increased as the aspect ratio of the fiber increased up to 500 [36]. However, when the starting pH of the reaction increased above 2.7 to obtain the HANF with a higher aspect ratio, the reactants were not completely dissolved. Therefore, it was difficult to synthesize HANF with an aspect ratio higher than 100.

**Figure 3:**
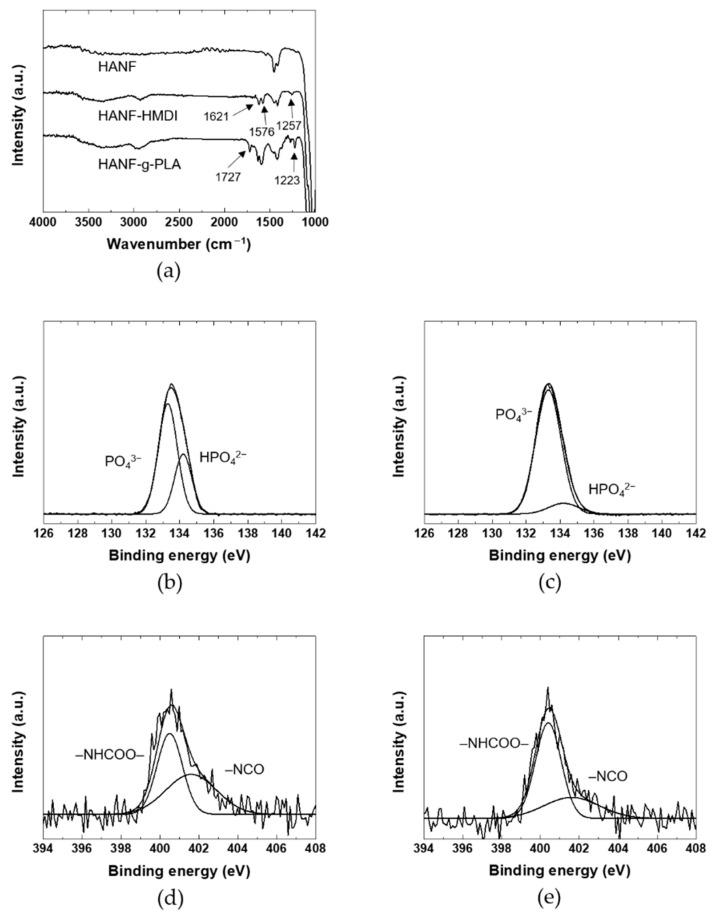
(**a**) FT-IR, (**b**) and (**c**) P2p XPS spectra, (**d**) and (**e**) N1s XPS spectra of HANF: (**b**) HANF; (**c**,**d**) HANF-HMDI; (**e**) HANF-g-PLA.

In addition to the aspect ratio of HA, the mechanical properties of PLA/HA composites were also affected by the dispersion of HA and the interface between HA and PLA. In order to improve the dispersion of HANF and interfacial adhesion between HANF and the PLA matrix, pre-synthesized PLA was grafted to HANF through HMDI as a coupling agent. HANF, HANF-HMDI, and HANF-g-PLA were investigated by FT-IR to identify the chemical bonds between HANF, HMDI, and PLA as shown in Figure 3a. The spectrum of HANF-HMDI was similar to that of HANF except for the characteristic peaks formed by HMDI. The spectrum exhibited the new peaks at 1621, 1576, and 1257 cm^−1^ due to the stretching of carbonyl groups, the bending of –NH, and the stretching of C–O–C in urethane bonds, respectively, which were formed by the reaction between the surface –OH groups on HANF and isocyanate groups in HMDI. The same bands were observed for polyurethane [37]. The bottom spectrum was for HANF-g-PLA. This spectrum was similar to that of HANF-HMDI except for new bands at 1727 and 1223 cm^−1^. The bands were assigned to the stretching of carbonyl groups and the stretching of C–O in ester bonds, which were formed by the reaction of the isocyanate groups in HANF-HMDI and the hydroxyl groups in PLA. The same bands were observed for the HA grafted by PLA [19].

In order to investigate the chemical bonds between HANF, HANF-HMDI, and HANF-g-PLA in detail, the XPS spectra of the HANFs were investigated as shown in Figure 3b–e. The P2p spectrum of HANF exhibited the peaks at 133.3 and 134.2 eV due to the PO_4_ and HPO_4_, respectively. The same bands were observed at titanium in electrolyte [38]. In the P2p spectrum of HANF-HMDI, the atomic ratio of PO_4_ to HPO_4_ was different to that of HANF. In the reaction of HANF and HMDI, the urethane bonds were formed by the reaction between the isocyanate groups in HMDI and the –POH groups in HPO_4_ [39]. The intensity of the peak associated with HPO_4_ at 134.2 eV decreased, and a new peak contributed by P–O–C bonds appeared approximately the same position as the peak of PO_4_ [40]. Although the –POH groups reacted by forming urethane bonds, the peak assigned to HPO_4_ did not disappeared completely. It could be explained that the peak of HPO_4_ present inside was obtained during XPS measurement. Or it might be because the density of –POH groups was higher than that of HMDI bonded at the highest density, leaving unreacted surface –POH groups.

In the N1s XPS spectrum of HANF-HMDI, the urethane bonds and the remaining isocyanate groups appeared at 400.5 and 401.6 eV, respectively. The same peaks were observed at the expanded graphite reacted with isocyanate groups [41]. It was found that side reactions such as dimerization of diisocyanate were avoided through the fact that the atomic ratio of the urethane groups to the isocyanate groups was approximately 1:1. In the N1s spectrum of HANF-g-PLA, the atomic ratio of the urethane groups to the isocyanate groups increased than that of HANF-HMDI due to the formation of the additional urethane bonds between the isocyanate groups in HANF-HMDI and the hydroxyl groups in PLA. Since the size of HMDI was smaller than that of the PLA, the peak attributed to the isocyanate groups still remained in the spectrum of HANF-g-PLA. In other study in which HA was reacted with an isocyanate coupling agent, the chemical bonds between the surface –OH groups on HA resulting from HPO_4_ and the isocyanate groups of HMDI have not been investigated in detail [42,43].

**Figure 4:**
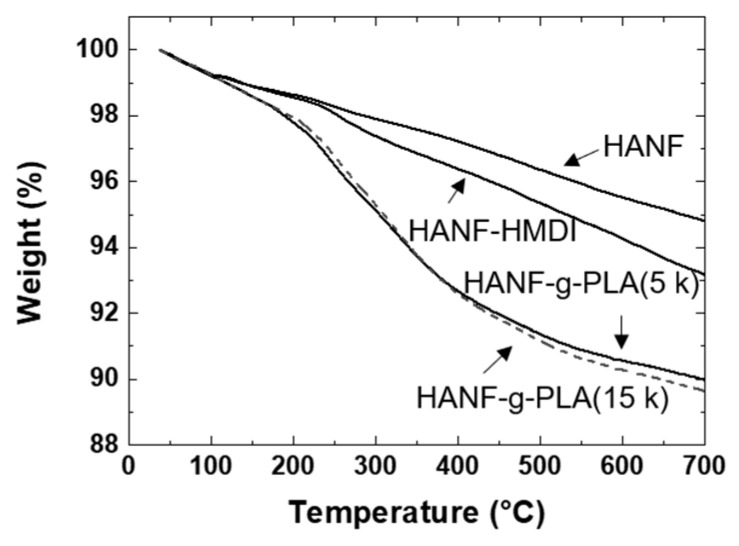
TGA of HANF, HANF-HMDI, HANF-g-PLA.

Since the amount, length, and frequency of grafted PLA would affect the interface between the matrix PLA and the HANF in the composites, it was necessary to perform the quantitative analysis on the grafted PLA. The amount of PLA grafted to HANF was investigated by TGA as shown in Figure 4. When grafting PLA with molecular weights of 5000 (HANF-g-PLA (5 k)) and 14,600 (HANF-g-PLA (15 k)), the weight loss of TGA was 3.2% and 3.5%, respectively. The end-to-end distance of the grafted polymer with the characteristic ratio of PLA of 11.8 was calculated as 7 nm and 12 nm, respectively, at theta condition [44]. When calculated based on the TGA results, molecular weight of the grafted PLA, Avogadro’s number, and the specific surface area of HANF of 52 m^2^/g, the grafted PLA had a chain every 13 nm^2^ and 36 nm^2^, respectively. The amount of PLA grafted to HANF was similar in both cases, as the larger polymers were grafted with lower frequency and the smaller polymers were grafted with higher frequency. Given the ratio of the length to frequency of the grafted PLA, it might be difficult to further graft the PLA to HANF in both cases.

The mechanical properties of the PLA/HANF-g-PLA composites were affected by the interface between the matrix PLA and the grafted PLA on HANF. Qiu et al. investigated the tensile strength of the PLA composites containing the HA grafted by PLA with different length [19]. The tensile strength of PLA resin was 63 MPa, and that of composites containing 10 wt% HA grafted with longer PLA and shorter PLA increased to 64 and 66 MPa, respectively. The difference in tensile strength of the composites was small because the degree of improvement of the interface between HANF and PLA of the composites was similar. It was assumed that the mechanical strength of the composite would be similar unless the length and frequency of the grafted chain were significantly changed. Even in the composites containing HANF-g-PLA(5 k) and HANF-g-PLA(15 k), the mechanical properties of the composites were not expected to differ significantly. Therefore, all subsequent experiments were performed with HANF-g-PLA(5 k). 

### 3.2. Mechanical Properties and Morphology

The tensile properties and the flexural properties of the composites were shown in Table 1. PLA/The HANF-g-PLA composites were labelled by the aspect ratio of HANF, the molecular weight of the grafted PLA, and compositions of filler. The symbols, (s) and (l), after HANF referred to HANF’s the aspect ratio of 70 and 100, respectively. The number after fillers denoted the components of fillers in wt%. For example, PLA/HANF(l)-g-PLA10 referred to the PLA composite containing 10 wt% of the HANF-g-PLA with aspect ratio of 100.

The tensile strength of PLA/HA(rod) was lower than that of PLA due to early fracture of the specimens in all compositions. In contrast, the tensile strength of PLA/HANF(s)5 was higher than that of PLA even though the interfacial adhesion between HANF and PLA was not improved compared to that between HA(rod) and PLA. This was because the external stress applied to the short fiber composites in tensile deformation was not concentrated only on crack growth due to the bridging effect of fiber [21].

The tensile strength of the composites was expected to increase further in composites using fibers with a higher aspect ratio. In order to investigate the effect of the aspect ratio of HANF on the tensile strength of PLA/HANF composites, the tensile strength of PLA/HANF(s)5 and PLA/HANF(l)5 was investigated. It was as expected that the tensile strength of the composite with a higher aspect ratio of fibers was higher than that of the composite with a lower aspect ratio of fiber. After this, all experiments were conducted with HANF(l). As HANF content increased, Young’s modulus of the PLA/HANF(l) increased compared to that of PLA resin. Despite the poor interfacial adhesion between the HANF and PLA, the failure of specimens was delayed due to the bridging effect of the fibers, resulting in increased tensile strength of the PLA/HANF(l) until 10 wt% of HANF(l) compared to that of PLA. The elongation at break of PLA/HANF(l)5 was higher than that of PLA/HANF(l)10, so the tensile strength was the highest in PLA/HANF(l)5. The Young’s modulus and tensile strength of PLA/HANF(l)5 increased by 38% and 13%, respectively, compared to those of PLA resin.

The elongation at break of PLA/HANF(l)-g-PLA was higher than that of PLA/HANF(l) for all compositions due to the entanglements between the graft PLA and the matrix PLA, resulting in higher tensile strength of PLA/HANF(l)-g-PLA than that of PLA/HANF(l). The tensile strength of PLA/HANF(l)-g-PLA was the highest in PLA/HANF(l)-g-PLA10, and the Young’s modulus and tensile strength of PLA/HANF(l)-g-PLA10 increased by 59% and 19% compared to those of PLA, respectively.

**Figure 5:**
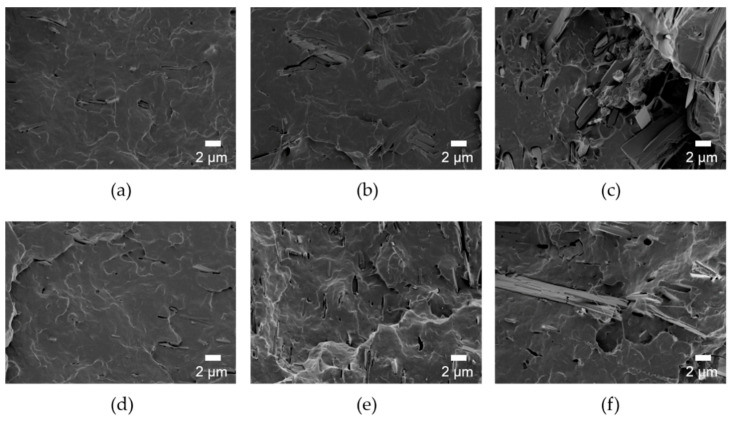
SEM images of fracture surface of (**a**) PLA/HANF(l)5, (**b**) PLA/HANF(l)10, (**c**) PLA/HANF(l)15, (**d**) PLA/HANF(l)-g-PLA5, (**e**) PLA/HANF(l)-g-PLA10, and (**f**) PLA/HANF(l)-g-PLA15.

Since the mechanical strength of the composites was also affected by the dispersion of the filler, the fracture morphology of the composites was investigated as shown in Figure 5. When the HANF was 10 wt% or more, the HANF aggregated in the composites. The composites were broken early because the stress was concentrated on the defects at the interface formed by the agglomeration of the HANF and the poor interfacial adhesion between the matrix PLA and the HANF. The dispersion of the HANF was improved by grafting PLA to the HANF due to the reduction of cohesion between the HANFs. Since Young’s modulus was determined at a very low deformation stage, Young’s modulus of the composites was affected by the dispersion of the filler [45]. For all compositions, Young’s modulus of PLA/HANF(l)-g-PLA was higher than that of PLA/HANF(l). The tensile strength and elongation at break of the composites were also affected by the dispersion of the HANF. More stress transfer between HANF(l)-g-PLA and PLA matrix occurred during tensile deformation through the improved interface.

For spinal implant materials, the flexural strength of the composites should be similar to that of human spine bone because the stress state experienced by spine bone was close to bending deformation rather than tensile deformation [46]. The tendency of changes in flexural properties of the composites was the same to that in tensile properties. Like tensile properties, flexural properties such as flexural modulus, flexural strength, and flexural strain at break were all higher for PLA/HANF(l)-g-PLA than PLA/HANF(l). In PLA/HANF(l)-g-PLA10, the flexural strength increased by 25% compared to that of PLA resin. Grafting PLA to the HANF though the coupling agent had a great influence on improving dispersion of the HANF and interfacial adhesion between the matrix PLA and the HANF. The increase in mechanical strength was much higher than that of previous PLA/HA particles composite studies [18,19,20,47,48]. The Young’s modulus of the PLA/HANF(l)-g-PLA10 was 4.8 GPa. Since the Young’s modulus of human compact bones has been known to be 9.8–15.7 GPa, the stress shielding effect might not occur in PLA/HANF(l)-g-PLA10 [49]. In terms of modulus of elasticity, PLA/HANF(l)-g-PLA10 appeared more suitable to be applied as spinal implant materials than metals.

### 3.3. Bioactivity

**Figure 6:**
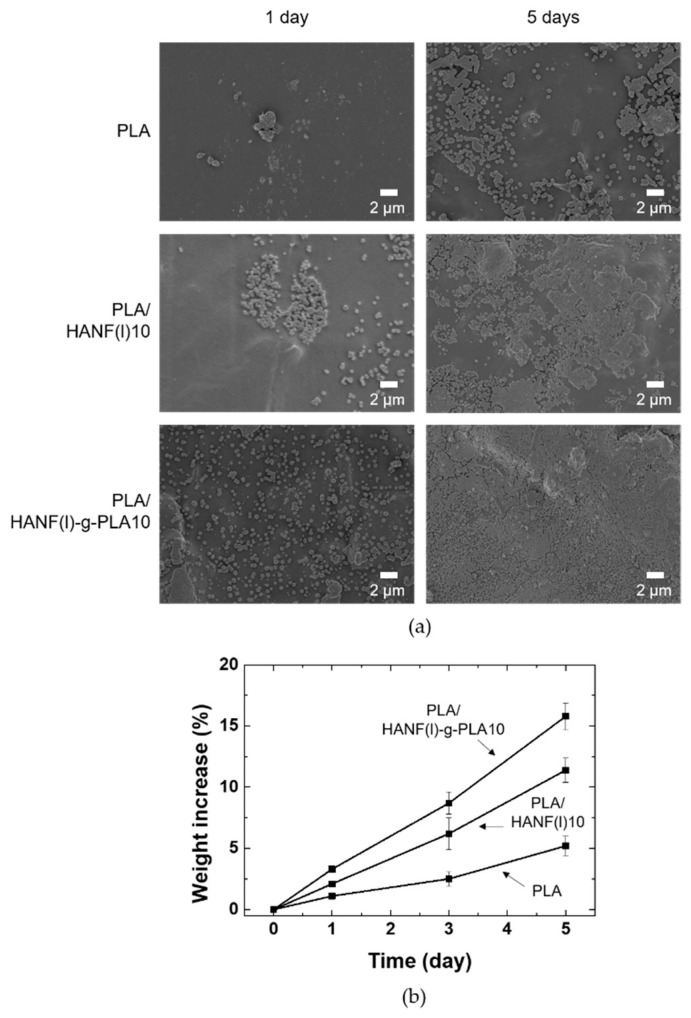
(**a**) Surface SEM images (**b**) weight increase of PLA and its composites after soaking in 5×SBF.

The bioactivity of PLA/HANF composites was studied to interpret bone recovery ability. Neat PLA, PLA/HANF(l)10, PLA/HANF(l)-g-PLA10 specimens were immersed in 5×SBF solution to observe the growth of new apatite on the specimen surface. The surface morphology of PLA and its composites immersed in 5×SBF solution was shown in Figure 6a. More apatite precipitated on the surface of PLA and its composites with immersion time. After soaking in 5×SBF solution for one day, the PLA specimen showed little growth of apatite on the surface, whereas the surface of PLA/HANF(l)10 was covered with more apatite particles. Since the nucleation of the new apatite originated from the HA on the surface, the PLA regions without the HANF still appeared to be clean even in PLA/HANF(l)10 [50]. The apatite crystals precipitated uniformly on the surface of the PLA/HANF(l)-g-PLA10 specimens compared to other specimens due to the large number of nucleation sites with the uniform dispersion of the grafted HANF. After 5 days of immersion, the surface of PLA/HANF(l)-g-PLA10 was almost covered with new apatite. The PLA specimen still showed a clean PLA surface area around the precipitated mineral, indicating that apatite was nonuniformly deposited on the neat PLA surface.

Figure 6b showed the weight increase due to the apatite formed by precipitation on PLA, PLA/HANF(l)10, and PLA/HANF(l)-g-PLA10 specimens in 5×SBF solution. The calculation of weight increase by apatite growth was based on the weight loss of the specimen in PBS solution over the same period [48]. Similar to the SEM images in Figure 6a, the weight increase was the least in PLA due to the small number of nucleation sites. The weight increase with the growth of the new apatite was greatest in PLA/HANF(l)-g-PLA10 because of the uniformly dispersed HANF. The newly grown apatite again acted as nucleation sites. This was also explained by the increase in rate of weight gain with immersion time for all types of specimens.

The PLA/HANF-g-PLA composite with improved bioactivity was manufactured in this study. In particular, the mechanical strength of this composite increased significantly compared to that of the PLA/HA particle composites in previous studies. However, the flexural strength of the composite needed to be further improved for the composite to be used as a spinal implant material because the flexural strength of human compact bones has been known to be 104–225 MPa [51].

## 4. Conclusions

HA was synthesized as nanofibers rather than particles. The starting pH of the reaction was changed to obtain higher aspect ratio HANF. HANFs with the aspect ratio of 70 and 100 were obtained at the reaction starting pH of 2.2 and 2.7, respectively. The tensile strength was also higher in PLA composites containing higher aspect ratio of HANF. PLA is grafted to further increase the mechanical strength of the composite, improving the dispersion of HANF and the interface between HANF and PLA. It was confirmed that even if the molecular weight of the grafted polymer was changed, the amount of grafted was similar. The tensile strength and the flexural strength of the PLA/HANF-g-PLA composites increased compared to those of PLA and PLA/HANF due to the reinforcement by HANF and improved interfacial adhesion by entanglement between the grafted PLA chain and matrix chain. In bioactivity test, new apatite grew faster in the composites containing HANF-g-PLA.

## Figures and Tables

**Table 1:** Mechanical properties of PLA and its composites.

	Young’s Modulus (GPa)	Tensile Strength (MPa)	Elongation at Break (%)	Flexural Modulus (GPa)	Flexural Strength (MPa)	Flexural Stain at Break (%)
PLA	2.9 ± 0.1	72 ± 2	3.4 ± 0.2	3.3 ± 0.1	99 ± 2	3.9 ± 0.1
PLA/HA(rod)5	3.3 ± 0.2	61 ± 4	2.0 ± 0.2	3.7 ± 0.2	81 ± 4	2.3 ± 0.2
PLA/HA(rod)10	3.5 ± 0.2	54 ± 3	1.7 ± 0.3	4.3 ± 0.3	74 ± 5	1.8 ± 0.1
PLA/HA(rod)15	4.0 ± 0.3	40 ± 3	1.1 ± 0.2	4.8 ± 0.2	65 ± 5	1.4 ± 0.2
PLA/HANF(s)5	3.8 ± 0.1	77 ± 3	2.2 ± 0.2	4.5 ± 0.3	107 ± 3	2.6 ± 0.1
PLA/HANF(l)5	4.0 ± 0.2	81 ± 2	2.4 ± 0.2	4.8 ± 0.2	112 ± 4	2.7 ± 0.2
PLA/HANF(l)10	4.6 ± 0.1	79 ± 2	1.9 ± 0.1	5.9 ± 0.2	101 ± 3	2.0 ± 0.2
PLA/HANF(l)15	5.0 ± 0.2	71 ± 3	1.4 ± 0.3	7.0 ± 0.3	92 ± 5	1.6 ± 0.3
PLA/HANF(l)-g-PLA5	4.1 ± 0.2	83 ± 3	2.6 ± 0.2	5.0 ± 0.2	116 ± 5	2.9 ± 0.3
PLA/HANF(l)-g-PLA10	4.8 ± 0.3	86 ± 2	2.3 ± 0.1	6.2 ± 0.3	124 ± 4	2.5 ± 0.2
PLA/HANF(l)-g-PLA15	5.1 ± 0.2	79 ± 3	1.7 ± 0.2	7.2 ± 0.3	109 ± 5	1.8 ± 0.3

## Data Availability

The data presented in this study are available on request from the corresponding author.
